# Chromogenic *Escherichia coli* reporter strain for screening DNA damaging agents

**DOI:** 10.1186/s13568-021-01342-1

**Published:** 2022-01-06

**Authors:** Josué Daniel Mora-Garduño, Jessica Tamayo-Nuñez, Felipe Padilla-Vaca, Fátima Berenice Ramírez-Montiel, Ángeles Rangel-Serrano, Fernando Santos-Escobar, Félix Gutiérrez-Corona, Itzel Páramo-Pérez, Fernando Anaya-Velázquez, Rodolfo García-Contreras, Naurú Idalia Vargas-Maya, Bernardo Franco

**Affiliations:** 1 Departamento de Biología, División de Ciencias Naturales y Exactas, Universidad de Guanajuato, Noria Alta S/N, 36050 Guanajuato, Gto Mexico; 2 Departamento de Microbiología y Parasitología, Facultad de Medicina, UNAM, Mexico City, Mexico

**Keywords:** DNA damage, Reporter strain, Chromogenic response, AmilCP, Environmental samples, Chromium pollution

## Abstract

The presence of pollutants in soil and water has given rise to diverse analytical and biological approaches to detect and measure contaminants in the environment. Using bacterial cells as reporter strains represents an advantage for detecting pollutants present in soil or water samples. Here, an *Escherichia coli* reporter strain expressing a chromoprotein capable of interacting with soil or water samples and responding to DNA damaging compounds is validated. The reporter strain generates a qualitative signal and is based on the expression of the coral chromoprotein AmilCP under the control of the *recA* promoter. This strain can be used simply by applying soil or water samples directly and rendering activation upon DNA damage. This reporter strain responds to agents that damage DNA (with an apparent detection limit of 1 µg of mitomycin C) without observable response to membrane integrity damage, protein folding or oxidative stress generating agents, in the latter case, DNA damage was observed. The developed reporter strain reported here is effective for the detection of DNA damaging agents present in soils samples. In a proof-of-concept analysis using soil containing chromium, showing activation at 15.56 mg/L of Cr(VI) present in soil and leached samples and is consistent with Cr(III) toxicity at high concentrations (130 µg). Our findings suggest that chromogenic reporter strains can be applied for simple screening, thus reducing the number of samples requiring analytical techniques.

**Supplementary Information:**

The online version contains supplementary material available at 10.1186/s13568-021-01342-1.

## Key points

- Reporter strains have become a relevant tool for environmental sample analysis.
- Chromoproteins are easy to detect reporter proteins.
- *recA*-AmilCP expression is easy to detect reporter strain for assessing DNA damage.

## Introduction

The increased anthropogenic activity has led to the accumulation of xenobiotics in water and soil. There is no industrial process that is 100% efficient, causing that all human activities generate pollution. Industrial activity has driven the introduction of pollutants in the environment that can affect resource usefulness and compromise human and animal health (Hill 2010). The most critical contaminants due to their effect on health are those generating DNA lesions such as metal ions, xenobiotics, and ultraviolet light.

The availability of metals in water and soil allows them to enter the body by food and water consumption. When metals reach vertebrate organisms, they become a risk factor for diseases. Some of them interact and form coordination complexes with nucleic acids, forming coordination complexes with nucleic acids, causing various DNA lesions (Theophanides and Anastassopoulou 2017).

Metals are naturally present in the soil, releasing erosion and reaching water sources. However, some industrial wastes are discarded in soil and water, increasing their concentration to hazardous levels (Hill 2010), highlighting the relevance of monitoring, and controlling their presence in the environment by assessing soil and water quality.

One example of a highly toxic heavy metal is chromium (Cr), which exists in oxidation states from − IV to + VI, showing high toxicity and posing a threat to human health (Saha et al. 2011). Cr is present in different sources, such as erosion, volcanic activity, mining activities, pigment production, stainless steel production, chrome plating, leather tanning, and other anthropogenic activities, that produces the highly toxic and mobile Cr(VI) (Saha et al. 2011). Detection and strategies for efficient removal of this contaminant is needed, for example, the use of biosorbents, to prevent the toxicity observed in living organisms that affect natural ecosystems (Saha and Orvig 2010).

Reporter strains developed in *E. coli* have been used for determining the dynamics in ecotoxicology due to the in-depth knowledge of this organism (Robbens et al. 2010). *Escherichia coli* is not an ideal reporter strain due to low resistance to the harsh conditions in polluted water and soil compared with other organisms such as *Pseudomonas*, *Acinetobacter,* or *Bacilli* species that resist these conditions. In the case of DNA damage, organisms showing higher resistance to DNA damage render poor reporters for assessing this kind of damage due to better repair mechanisms. Also, several molecular tools are available for *E. coli* that generate this organism a highly flexible chassis for developing reporter strains.

In the present study, we developed and characterized a qualitative reporter strainusing a recombinant *Escherichia coli* that express the coral *Acropora millepora* chromoprotein (AmilCP) under the control of the *recA* promoter sequence. This strain shows activation with exposure to DNA damaging agents. The *recA* gene in wild-type *E. coli* cells is expressed at a low rate during exponential growth due to repression by LexA protein. In cells exposed to DNA damaging treatments, the interaction of RecA with ssDNA activates its protease activity, facilitating the autocatalytic cleavage of the LexA repressor and allowing the *recA* gene transcription. Under DNA damaging conditions, the rate of RecA protein production is around tenfold higher than during normal growth (Little et al. 1981).

The bioreporter strain presented in this work can be applied to discriminate samples that need further analytical testing. By using a straightforward methodological approach by placing a mat of cells on top of LB-agar plates, the reporter strain shows reduced cross-reactivity. This design is promising for its application since no sample modification or preparation is needed for analysis, and the result is easy to interpret. As a proof of concept, the strain is tested with soil and water samples containing the environmental contaminant Cr(VI). The results presented here add to the existing bacterial reporter strains designed for genotoxic detection.

## Materials and methods

### Strains used and growth conditions

*Escherichia coli* MG1665 strain (obtained from the Coli Genetic Stock Center, Yale University, USA) was used in this study for in vivo experiments. JM109 strain (Promega, WI, USA) was used for plasmid construction and propagation. *Escherichia coli* cells were grown in Lysogeny Broth (LB) medium at 37 °C. For cells bearing plasmid pQE30 (Qiagen, Hilden, Germany) or pRecA-AmilCP (this work), LB medium was supplemented with ampicillin to a final concentration of 200 µg/mL.

### Plasmid construction

The *recA* promoter sequence was amplified from the wild-type MG1665 strain (gene accession number KEGG b2699) with the following primers *recAamilXho*I_Fw (5′-ACTGCTCGAGAGAGAAGCCTGTCGGCAC-3′) and *recAamilBam*HI_Rv (5′-CACGGATCCCATTTTTACTCCTGTCATGCCG-3′) using the following amplification conditions: 30 s at 94 °C, 45 s at 50 °C and 50 s at 72 °C, 34 cycles. By using standard molecular biology techniques, the *recA* promoter fragment was ligated into the pQE30 plasmid (Qiagen, Hilden, Germany) which has the *amilCP* gene sequence (iGEM accession number BBa_K592009) cloned as *Bam*HI-*Hin*dIII fragment (Tafoya-Ramírez et al. 2018). All restriction enzymes were purchased from New England Biolabs (Beverly, MA, USA). XL1-Blue MRF' (Agilent, Santa Clara, USA) and MG1665 *E. coli* strains were transformed with the resulting plasmid pRecA-AmilCP. The final cassette with the *amilCP* gene under the control of *recA* promoter was tested by colony PCR with the primers recAamil*Xho*I_Fw and amilColi*Hin*dIII_Rv (5′-ACCAAGCTTTCATTAAGCAACAACCGGC-3′) and the following amplification conditions: 30 s at 94 °C, 30 s at 47 °C and 1 min at 72 °C, 34 cycles. The PCR reactions were performed with GoTaq IX Green Master mix (Promega, WI, USA).

### Plate assay for reporter strain activation

Cells transformed with the plasmid pRecA-AmilCP were grown in LB liquid media for 16 h. 150 µL of cell culture was mixed with LB soft agar and poured on LB agar plates. Sterile filter disks were placed on the top of LB agar and were used to place mitomycin C (MC) diluted in water (0.015, 0.1, 0.15, 0.5, 1, 1.5, 2, 2.5, and 3 µg/mL) and showed both the qualitative response and the plot of halo activation diameter and MC concentration used. Also, as part of the characterization, different damaging agents: ethanol (1.5, 3, 5, 6.5, 10 and 15%), NaCl (0.5 M), SDS (0.3, 0.7, 0.8 and 1%) and H_2_O_2_ (1, 4, 6, 8 and 10 mM) using the same volume of each agent throughout (3 µL). Plates were incubated at 37 °C for 18 h. Sterile water was used as a negative control. Additionally, an LB agar plate with bioreporter mat but without discs was used for UV light exposure (5000 and 10,000 µJ/cm^2^, Stratalinker 2400, Stratagene, La Jolla, CA) using an aluminum foil to cover the plate except for a 5 cm × 1 mm slit to expose cells.

For measuring the effect of reactive oxygen species generated by the Fenton reaction, LB agar was supplemented with Fe (NH_4_)_2_(SO_4_)_2_ (0.1 and 1 mM), and H_2_O_2_ (1, 6, 8, and 10 mM) was placed on the disks. MC (0.75 µg) was used as a positive control for this assay. Plates were incubated for 24 h at 37 °C.

### Cell viability determination

These assays were performed by growing cells for 16 h and adjusting cell cultures to an O.D._600_ of 0.5. Cells from 1 mL of culture were recovered by centrifugation, resuspended in 500 µL of MC (0.15 and 3 µg), and incubated 60 min at 37 °C with agitation. Cells were centrifuged and resuspended in 500 µL of PBS. PBS was used as a negative control. For the heat shock assay, cells were resuspended on PBS and incubated for 30 min at 50 °C or 37 °C for the control. Serial dilutions of each cell suspension were spotted on LB agar (3 µL) and were incubated for 16 h at 37 °C.

#### Cells in suspension tests for reporter activation

Cells containing the plasmid pRecA-AmilCP were grown to an O.D._600_ of 0.5. Cells from 1 mL of culture were recovered by centrifugation, resuspended in 500 µL of H_2_O_2_ (1, 5, and 10 mM), and incubated for 1 h at 37 °C with agitation. PBS was used as a negative control. In the heat shock test, cells were resuspended in PBS and incubated for 30 min at 50 °C or 37 °C for the control. After the incubation time, cells were centrifuged in both assays, and the supernatant was discarded.

### Reporter strain performance with Cr(III) and Cr(VI) reference solutions

We followed the disk assay protocol to test the effect of Cr(VI) and Cr(III) on the reporter strain. We added K_2_CrO_7_ (0.017, 0.17, 1.7 and 17 µg) and CrCl_3_ (2.5, 50.0 and 130.0 µg) for the Cr(VI) and Cr(III) respectively. Additionally, we performed the assays without disks by spotting K_2_CrO_7_ (0.3, 3, and 30 µg) and CrCl_3_ (0.25, 0.3, and 30.0 µg) solutions directly on the plate.

For testing DNA damage due to the presence of Cr(VI), soil and leached contaminated samples were obtained from a chromite-processing industry in central Mexico previously characterized (Piñon-Castillo et al. 2010) and containing 7040 and 5100 mg/g of total chromium during the dry and wet seasons respectively. The content of Cr(III) and Cr(VI) in the sample, for which we determined the total chromium content by Flame Atomic Absorption Spectrometry (ETA-AAS) and Cr(VI) content by UV–Vis spectrophotometry using diphenyl carbazide (Greenber et al. 1981). The Cr(III) content was determined by subtracting from the total Cr content (4632.4 mg/L) the Cr(VI) content (3890.25 mg/L), which gives the amount of 742.15 mg/L of Cr (III). This result indicates that in the industrial waste leachate sample used in the experiments, the majority of the chromium content (83.97%) corresponds to Cr(VI), with a lower proportion (16.03%) of Cr(III). For the lixiviates samples, lixiviate 1 from a contaminated source and lixiviate 2 from a control sample, we followed the disk-diffusion assay. Different lixiviate concentrations (direct sample, 1:5, 1:10, and 1:100 dilutions) were used for the assay. Water was used as negative control and for diluting the lixiviates. Instead of placing filter disks for the soil samples, 5 mg of soil were placed directly on the LB plates, and each soil fragment was covered with 25 µL of LB soft agar.

All the experiments were conducted in triplicates using independent replicates; representative plates are shown.

## Results

### Reporter strain generation and characterization

**Fig. 1:**
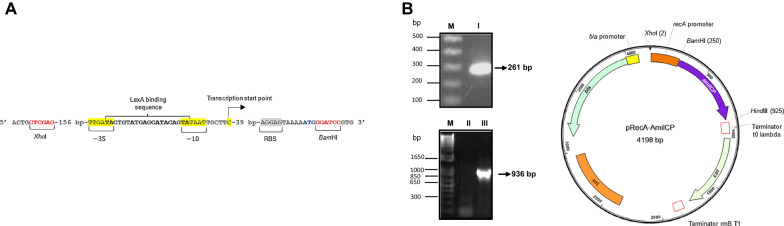
Reporter plasmid map used in this study. **A** elements present in the *recA* promoter sequence used. **B** plasmid map and PCR confirmation of the insert of the promoter sequence (261 bp amplicon) and promoter and AmilCP coding sequence PCR verification (936 bp PCR amplicon). Restriction sites are indicated and in parenthesis the site of cleavage

To construct a DNA damage reporter strain, we used the *recA* promoter region that is sufficient for driving a reporter gene expression and suitable for assessing DNA damage from chromosomal integrations or plasmids reported previously (Vollmer et al. 1997; Rosen et al. 2000) (Fig. 1A).

The *recA* PCR amplified promoter sequence (261 bp) was cloned into a plasmid containing the coding sequence for AmilCP chromoprotein. The correct cloning of the *recA* promoter sequence was PCR-confirmed by using the forward promoter primer and the AmilCP coding sequence reverse primer (Fig. 1B); confirming the correct cloning into a plasmid with the stable and less toxic AmilCP reporter (Tafoya-Ramírez et al. 2018; Liljeruhm et al. 2018). This construct contains the plasmid backbone pQE30 that has the pBR322 replication origin, rendering ~ 40 copies per cell (plasmid map in Fig. 1B) (Dykxhoorn et al. 1996), thus preventing a titration effect of LexA that may result in higher levels of background response. We introduced the resulting plasmid pRecA-AmilCP into *E. coli* cells to generate a DNA damage reporter strain.

Strain validation with the plasmid pRecA-AmilCP were done as follows. Cells were exposed to different concentrations of mitomycin C (MC). This antibiotic generates crosslinks between DNA strands from other types of cells (Iver and Szybalski 1963). The reporter strain response to DNA damage caused by MC was evidenced by a purple halo around the disk where MC was applied over the mat of the reporter cells. As a control, *uspA* promoter sequence was used in the same plasmid instead of the *recA* promoter to show that the activation color is due to the expression of AmilCP and not the MC used to generate DNA damage (Additional file 1: Fig. S1A).

**Fig. 2:**
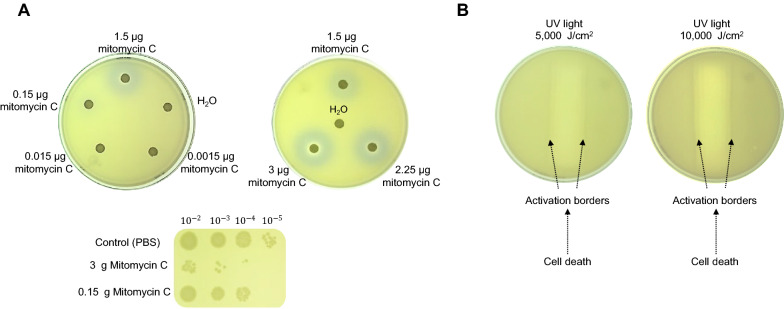
Reporter strain characterization with mitomycin C. **A** shows different concentrations of mitomycin C applied to the plate using sterile paper disks. Plates show low and high mitomycin C concentrations. In the lower panel, cell viability was assessed by spot assay shown at high and low concentrations of mitomycin C. The control spot contained sterile water. **B** shows the effect of UV light exposure through a 1 mm slit. Two UV-light intensities were used. The clear area on each experiment indicates cell death. The purple halos indicate reporter strain activation

With the concentrations tested using 35 mm plates and measuring the halo (Additional file 1: Fig. S1E), the activation halo is visible at 1 µg of MC. By plotting the halo diameter against the concentration, a concentration-dependent formation of the activation halo is observed (Additional file 1: Fig. S1E), with an observable limit at 1 µg of MC. At higher concentrations, these purple halos were accompanied by an inhibition growth area surrounding the paper disk or direct exposure to MC, which was also confirmed by viability assays (Fig. 2A).

Bacteria are highly susceptible to UV irradiation due to their small size, short generation time, and the absence of radiation protective pigmentation (Santos et al. 2013). DNA can absorb UV radiation, and the direct excitation of DNA produces cyclobutene pyrimidine dimers identified as the primary cause of mutagenesis and the activation of *recA* (Kielbassa et al. 1997). To analyze the effect of UV light on the reporter strain, we exposed the cell mat in a plate to different intensities of UV light. We observed a region where cell growth was inhibited, and the vicinity of the direct UV light exposure showed a purple color frame, indicating reporter strain activation. With a higher intensity of UV radiation, we observed a substantial reduction of growth in the direct exposure and a stronger purple frame, indicating reporter strain activation (Fig. 2B).

To test the reporter strain specificity, we analyzed the effect of different agents known to disturb cell components and biological processes in *E. coli* other than DNA damage.

**Fig. 3:**
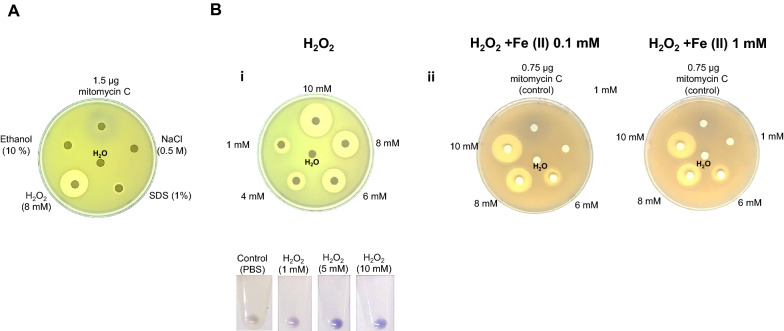
Reporter strain specificity test. **A** shows the effect of osmotic, membrane, oxidative, and protein stress (clockwise) using NaCl, SDS, H_2_O_2,_ and ethanol as the corresponding perturbing agents at the indicated concentration. **B** shows the effect of different concentrations of H_2_O_2_. **i** shows the effect of H_2_O_2_ without iron in the media, lower panel shows the transitory activation of the reporter strain after a 30-min exposure to different concentrations of H_2_O_2_. **ii** shows the effect of two concentrations of iron in the media and the effect of exposure to H_2_O_2_ at different concentrations emulating a Fenton reaction. The iron present in the media is indicated at the bottom of each image. The clear area on each experiment indicates cell death. The purple halos indicate reporter strain activation

Hyperosmotic stress (0.5 M NaCl) (Record et al. 1998) was used to test the reporter strain response, and no activation was detected; only a slight reduction in growth was observed (Fig. 3A).

The outer and cytoplasmic membranes of *E. coli* are sensitive to ionic detergents. We analyzed the reporter strain response to sodium dodecyl sulfate (SDS), an agent known to disrupt the cytoplasmic membrane and thus activate *fabA* (Woldringh and van Iterson 1972; Bechor et al. 2002). As expected, there was no detectable response of the reporter strain (Fig. 3A and for more concentrations tested, refer to Additional file 1: Fig. S1B).

Ethanol has been extensively used to simulate protein misfolding and in *E. coli* affects cell wall, and membrane integrity inhibits the protein synthesis. It can bind to proteins affecting their function (Ingram and Vreeland 1980; Dombek and Ingram 1984; Haft et al. 2014; Cao et al. 2017). We found no activation of the reporter strain when exposed to ethanol (Fig. 3A and for more concentrations tested, refer to Additional file 1: Fig. S1C).

Partially reduced reactive oxygen species (ROS) can affect the DNA integrity in cells (Imlay 2013) and thus is relevant to determine DNA damage in the presence of oxidant agents. We exposed the generated reporter strain to H_2_O_2_, showing growth inhibition in the plate-disk assay, which was more evident than the activation purple halo at the concentration used (Fig. 3A). When the experiment was carried out with cells in suspension, the activation of the reporter strain was evident by an increasing purple coloration at increasing concentrations of H_2_O_2_ under the same incubation time (Fig. 3Bi), which correlates with the rapid accumulation of peroxy-radicals inside the cell in the short term. To determine if peroxy-radicals in the plate assay could be generated, the Fenton reaction was used to produce hydroxyl radicals with Fe (II) addition into the LB agar plates. We detected that reporter strain activation was accompanied by grow inhibition area in a concentration-dependent manner (Fig. 3Bii). No significant differences were observed in either 1 or 0.1 mM Fe (II) concentrations.

The growth inhibition area is reduced probably to a reduction of H_2_O_2_ available in the media due to Fe oxidation, thus reducing its toxic effect. The activation halo is not significantly larger than the observed without Fe in the media. Still, the extent of a more blue-purple area surrounding the application spot may indicate more diffusion of H_2_O_2_, thus rendering an activation halo more diffuse.

Finally, as part of the reporter strain characterization, we tested the induction of *recA* during heat shock stress (Fabisiewicz and Janion 1992). We tested stressed cells by spot assay and transitory expression of *recA* in cells stressed in suspension. In Additional file 1: Fig. S1D, expression is not visible in cells only heat shocked for 30 min. and then plated by spot assay where no activation is expected since the damaging agent is tenfold diluted in the first spot and then serially diluted, but if cells are centrifuged after incubation with the damaging agent, apparent activation is detected in comparison with control cells where a light purple background is observed.

The MC detection limit is estimated in 1 µg MC applied to the plate as shown in Additional file 1: Fig. S1E, by direct measurement of the activation halo in 35 mm plates. This result is as sensitive as a previously described reporter strain using GFP (Padilla-Martínez et al. 2015).

### Reporter strain activation with chromium

Chromium compounds are considered highly toxic and mutagenic for cells (Fang et al. 2014). In bacteria, Cr(VI) quickly enters the cytoplasm, where it can be reduced to lower oxidation states [i.e., Cr(III)]. During this reduction process, free radicals may be formed, which can cause oxidative damage to DNA (Ramírez-Díaz et al. 2008).

**Fig. 4:**
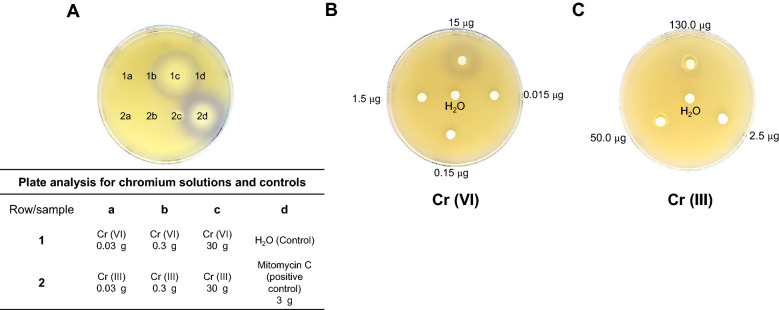
Reporter strain activation by chromium toxicity. In **A** the reporter strain was exposed to different concentrations of chromium solutions at two oxidation states, the highly toxic Cr(VI), and the less toxic form Cr(III). The table indicates the concentrations applied on each section of the plate. The experiments were carried applying directly 3 µL of each chromium solution. **B** shows the effect of lower concentrations of Cr(VI) and **C** shows higher concentrations of Cr(III). The clear area on each experiment indicates cell death. The purple halos indicate reporter strain activation

Here, we assessed the reporter strain response to increasing quantities of Cr(VI) and Cr(III) (Fig. 4A). When cells were exposed to Cr(VI), a purple halo of activation was evidenced at 30 µg (Fig. 4A). Lower concentrations showed that at 15 µg of chromium applied to the paper disc showed activation but lower than this in decimal dilutions showed no activation (Fig. 4B).

Cr(III) showed no reporter strain activation at low concentrations (Fig. 4A). Slight purple coloration was noticed when cells were exposed to higher concentrations of Cr(III) (Fig. 4C). This suggests that Cr(III) induce DNA damage at high concentrations.

These observations of the reporter strain indicate a genotoxic property of Cr(III), in agreement with the demonstrated mutagenicity and DNA damage in the yeast *Saccharomyces cerevisiae* (Fang et al. 2014).

### Reporter strain activation with environmental samples

To test the functionality of the reporter strain to detect agents that produce DNA damage and are present in soil and water samples, we performed a proof of concept using leachate and soil characterized samples containing chromium and other heavy metals in lower concentrations from a chromite-processing industry (Piñon-Castillo et al. 2010). We tested the performance of the reporter strain using either a paper filter disk or direct exposure of the samples to the cell mat to reduce sample analysis that surpasses the norm limit (ISO 23913:2006).

**Fig. 5:**
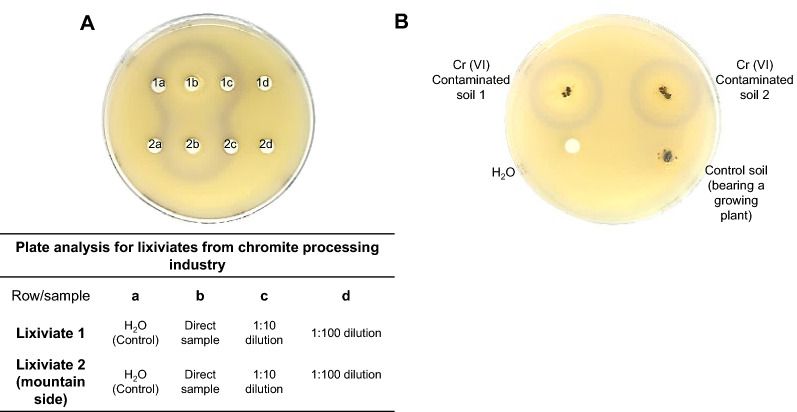
Reporter strain activation with samples from polluted soil from a chromite processing facility. In **A** the reporter strain activation was assessed with different concentrations of the leachate soil. The table indicates the concentration applied to the plate. In **B** the reporter strain was exposed to samples of soil directly placed on top of the soft agar-cell mat surface and fixed with soft LB-agar. As a control, soil from a growing plant was used. The clear area on each experiment indicates cell death. The purple halos indicate reporter strain activation

We observed a purple halo of activation when we applied two independent lixiviate contaminated soil samples to the reporter strain. This reporter strain response was strong using concentrated samples (Fig. 5A). Still, in the case of the lixiviate 1, the biosensor stimulation was detected with a 1:5 (v/v) dilution in a separate plate (Additional file 1: Fig. S2A). As a control, we tested a mountainside lixiviate with no chromium under the same conditions (Additional file 1: Fig. S2B), showing no activation of the reporter strain. The approximate final concentration of 15.56 mg/L for Cr(VI) in the plate (using 25 mL plates) and 2.96 mg/L final concentration for Cr(III) present in the sample.

Finally, we directly examined the reporter strain response, exposing two independent soil samples into the plate with the approximate final concentrations as stated above. We detected a purple halo around each soil sample, indicating the positive detection of DNA damaging compounds (Fig. 5B). Exposure to a soil sample from a growing plant shows no detectable activation of the reporter strain (Fig. 5B). The resulting concentrations of Cr(VI) and Cr(III) are above the norm and produce cell damage is Cr(VI) present in the sample, but by discriminating samples that activated the reporter strain, further analytical analysis done will be aimed to the samples lower than the detection threshold.

## Discussion

DNA damage has been studied using the single-cell gel electrophoresis technique or Tunel, which has been improved in sensitivity and the information obtained from this technique. In Tunel, mapping regions of interest (by coupling to Fluorescence In Situ Hybridization or FISH) shows low levels of damage (Mondal and Guo 2017). Recently, that system can be escalated to high throughput levels, as reported by Shaposhnikov and colleagues (Shaposhnikov and Collins 2017). Still, this approach only renders the amount of damaged DNA and requires sampling, specimen preparation, and analysis, and these actions require specialized personnel and costly equipment. Additionally, single-cell strategies cannot differentiate damage from xenobiotic agents that are not available due to their association with organic matter. The toolbox for assessing genotoxicity in different samples is ample (Johann et al. 2020), such as cell viability by MTT assays (Mosmann 1983), oxidative stressors by Nrf2-CALUX (Steinberg et al. 2016), micronucleus assay for chromosomal aberrations (ISO 21427-2) and the Ames mutagenicity assay (ISO 11,350).

The reporter strains intended for detecting chemical or physical changes in the environment contain a transducing signal module that produces a measurable signal or the use of logic circuits that connect cellular regulatory networks to a detectable output when the desired condition is met (D’Souza 2001). Reporter strains have become a novel tool for analytical applications and are now gaining interest in environmental sciences.

Bacteria have been recognized as valuable tools for ecotoxicological studies. They monitor several compounds identified as pollutants and health hazards by generating a specific response. They have served to quantify specific damage, for example, DNA damage during exposure to xenobiotic compounds or even explosives (Quillardet and Hofnung 1993; Vasilieva 2002; Sphigel et al. 2021; Zeng et al. 2021).

*Escherichia coli* is an organism that has been extensively studied in all aspects of its physiology (Vargas-Maya and Franco 2017). Recently, it has become an essential bioreporter due to the numerous molecules that have been detected using it as a host for reporter constructs (Robbens et al. 2010; Zeng et al. 2021). Although, limitations to its resistance to extreme conditions limit its use for studying complex samples such as soil and water.

Controversy regarding the use of engineered bacteria to monitor the presence of highly toxic compounds rises from the fact that dead cells cannot generate a response (Sørensen et al. 2006); for such reason, analytical methods are still the gold standard for environmental monitoring. Nonetheless, bioreporters provide a threshold for analyzing specific analytes by specific pathways and information regarding the generated cell stress (Sørensen et al. 2006).

Alternative approaches can be implemented using bacterial reporter strains. A bacterial reporter strain that emits a qualitative signal with a defined activation threshold could be important for sample screening. This may be a relevant tool for further analysis to discriminate samples with toxic compounds using analytical tools such as High-Performance Liquid Chromatography following regulatory norms (such as SR ISO 10304-1:2007). For this reason, bacteria are ideal tools for reducing the number of samples requiring extensive analytical testing. In turn, this will reduce the costs of environmental monitoring, and more importantly, the biological impact can be determined.

The reporter strain described here, with the ease of applying samples directly into the plate, two aspects can be determined. Cell death is observed where the concentration is high in the application spot. Once diluted by diffusion, the second aspect is the activation of the reporter strain, that canbe evidenced in the surrounding cell death zone and the 'on/off' response can provide information for fast decision making in a similar fashion as described by Zhang et al. (2013) or generate more portable and complementary tools for in-field detection (Roggo and van der Meer 2017). The choice of AmilCP is based on a previous study that showed that AmilCP is stable at a wide range of pH values and exposure to urea and an oxidant (Tamayo-Nuñez et al. 2020), thus making it suitable as a reporter under harsh conditions, such as contaminated water and soil samples (Jiang et al. 2017).

With the validation using common DNA damaging agents, the strain performance is acceptable for the specificity since no other cell-damaging compounds tested in this work showed activation of the reporter. The response is effective after 18–24 h. incubation. Samples can be applied directly to the cell mat, resulting in the release of the xenobiotic compound, here Cr (VI). Finally, the activation was consistent with MC or other DNA damaging compounds.

The activation of the reporter strain occurred with 15 µg of Cr(VI), which is 10 times higher than the accepted limit of 0.1 µg per mL for human consumption (ISO 23913:2006). Samples exciding this limit are classified as non-drinkable. Therefore, the reporter strain is an aid for discriminating and limiting the samples that need analysis with analytical tools.

Overall, the existing literature on reporter strains supports using a fast and reliable reporter strain such as the one reported here that can be easily detected, even that quantification is not straightforward with AmilCP due to the same absorbance as whole cells. Nevertheless, the results presented here suggest that the same principle as reported by other authors can be applied using chromoproteins. In future work, it can be used extensively in Cr contaminated soils.

Soil and water quality assessment needs careful sample collection, transportation to a laboratory facility equipped with the proper instrumentation, and highly trained personnel for sample preparation, analysis, and interpretation (Frentiu et al. 2013). This process is time-consuming, costly, and requires toxic reagents in some cases. Although showing the need for incubation time, the reported strain in this study significantly reduces the need for preservation of samples for transportation and can be applied directly in the field. Further improvements may be required, such as using more suitable organisms for testing contaminated water and soil samples. Still, as a proof-of-concept, the AmilCP reporter provided a specific response.

Several reports show that bacteria bearing a plasmid or chromosomal fusion of the *recA* promoter to the coding sequence of reporter proteins, are sensitive to DNA damaging agents and show an acceptable detection threshold. This type of reporter strains are fast and high throughput tools for screening soil and water samples (Woutersen et al. 2011; Vollmer et al. 1997; Padilla-Martínez et al. 2015; Chen et al. 2012; Martineau et al. 2009; Østergaard et al. 2007; Zeng et al. 2021). Also, *lacZ* is currently used as a reporter in reporter strains for determining mutagenicity in bacteria (Vasilieva 2002). A chromoprotein may expand the applications in other bacteria more suitable for environmental sampling (Song et al. 2009).

These systems are both specific and sensitive and can trace the extent of damage and correlate it to the concentration of the hazard (Ahn et al. 2009). With the development of reporter plasmids bearing either luminescent or fluorescent proteins, the results are comparable (Kohlmeier et al. 2007), with the disadvantage that these reporters need devices such as fluorometers or luminometers. In the case of *luxCDABE*, constructs are in the range of 10 kb, which is more unstable or challenging to transform.

Several novelties have been done in the design of reporter strain using *gfp*, *lacZ,* and *luc*/*lux* genes as reporters (Ghim et al. 2010) and continue to be the gold standard of reporters. Nevertheless, chromoproteins may be positioned as novel and efficient reporters. To our knowledge, there is only one example of the whole-cell reporter strain using a chromogenic reporter protein for pyrethroid insecticide exposure (Riangrungroj et al. 2019).

The results presented here show that the use of AmilCP chromoprotein renders a simple method for assessing the presence of DNA damaging compounds in an environmental sample. Previous uses of reporter strains, as reported by Vollmer (1998), where *recA::lux* fusion renders in-plate response by showing growth inhibition and reporter activation, in a similar fashion that the one showed here, with the advantage that no film or CCD camera is needed.

By no means this whole-cell reporter strain can substitute analytical methods. Still, it may reduce the number of samples needing analytical methods, thus reducing the costs, and generating hazardous materials during sample preparation. In previously reported strains using *lacZ* expression, high background activity was detected. Here the background is significantly reduced and showed equivalent sensitivity as a GFP reporter to MC (in the range of 1 µg of MC) (Padilla-Martínez et al. 2015).

The major disadvantage of AmilCP is the maximum absorbance of the protein at 589 nm (http://parts.igem.org/Part:BBa_K592009, Contribution: Valencia UPV iGEM 2018), which makes absorbance measurements using whole cells complicated, and further processing will be required before measuring the amount of AmilCP produced for quantitative analysis. Nevertheless, in the present study, the results suggest that using the *recA*-AmilCP reporter plasmid can render a fast and safe method for analyzing many samples. Also, this reporter strain was used to assess the microbicidal effect of phenothiazine derivatives causing cell death by damaging the cell membrane and DNA (Aguilar-Vega et al. 2021). The use of whole-cell reporter strain can help determine the primary cell effect of antibiotics and antimicrobial agents and further determine the improvements on current antimicrobials, repurposing drugs, and test newly discovered molecules for antimicrobial applications.

Further research is needed to generate a more versatile system for high throughput analysis, develop a consistent quantification method, or expand the design to other toxic compounds’ detection using a similar approach. In *E. coli*, for example, arsenic detection (van Genuchten et al. 2018) or the detection of complex compounds using heterologous enzymes and detection systems such as the reported for benzaldehyde detection (van Sint Fiet et al. 2006) or metabolite products (Rogers and Church 2016).

## Supplementary Information

**Additional file 1: Figure S1. **Additional controls used in this study. **Panel A **shows the expression of AmilCP under the control of the *uspA* (Universal Stress Protein) promoter sequence. As an activating agent, H_2_O_2_ was used and mitomycin C as a negative control (MC). **Panel B** shows the effect of varying concentrations of SDS on the activation of the *recA* reporter strain. **Panel C **shows the activation of varying concentrations of ethanol. **Panel D** shows the viability and activation of the reporter strain during a 30 min. heat shock. The clear area on each experiment indicates cell death. The purple halos indicate reporter strain activation. Panel E, representative examples of 35 mm petri dish assays using MC at different concentrations and the plot of halo diameter against MC concentration, an average of three independent experiments, to show that below 1 µg of MC the signal is barely detectable. **Figure S2. **Evaluation of lower concentrations of the chromium contaminated lixiviates from a chromite processing facility. **Panel A** shows the activation of the reporter strain at lower concentrations of the Cr(VI) containing lixiviates. In **Panel B**, control lixiviates are shown from the mountainside of the contaminated soil known to be clear of Cr(VI). The clear area on each experiment indicates cell death. The purple halos indicate reporter strain activation.

## Data Availability

The datasets generated for this study are available on request to the corresponding authors.
