# Supplementary material for: Chromogenic Escherichia coli reporter strain for screening DNA damaging agents
Source: AMB Express. 2022 Jan 6;12:2. doi: 10.1186/s13568-021-01342-1 (PMC8739417; doi:10.1186/s13568-021-01342-1)
Supplement: Supplementary file 1 — Additional file 1: Figure S1. Additional controls used in this study. Panel A shows the expression of AmilCP under the control of the uspA (Universal Stress Protein) promoter sequence. As an activating agent, H2O2 was used and mitomycin C as a negative control (MC). Panel B shows the effect of varying concentrations of SDS on the activation of the recA reporter strain. Panel C shows the activation of varying concentrations of ethanol. Panel D shows the viability and activation of the reporter strain during a 30 min. heat shock. The clear area on each experiment indicates cell death. The purple halos indicate reporter strain activation. Panel E, representative examples of 35 mm petri dish assays using MC at different concentrations and the plot of halo diameter against MC concentration, an average of three independent experiments, to show that below 1 µg of MC the signal is barely detectable. Figure S2. Evaluation of lower concentrations of the chromium contaminated lixiviates from a chromite processing facility. Panel A shows the activation of the reporter strain at lower concentrations of the Cr(VI) containing lixiviates. In Panel B, control lixiviates are shown from the mountainside of the contaminated soil known to be clear of Cr(VI). The clear area on each experiment indicates cell death. The purple halos indicate reporter strain activation. [file 13568_2021_1342_MOESM1_ESM.pptx]

## Slide 1
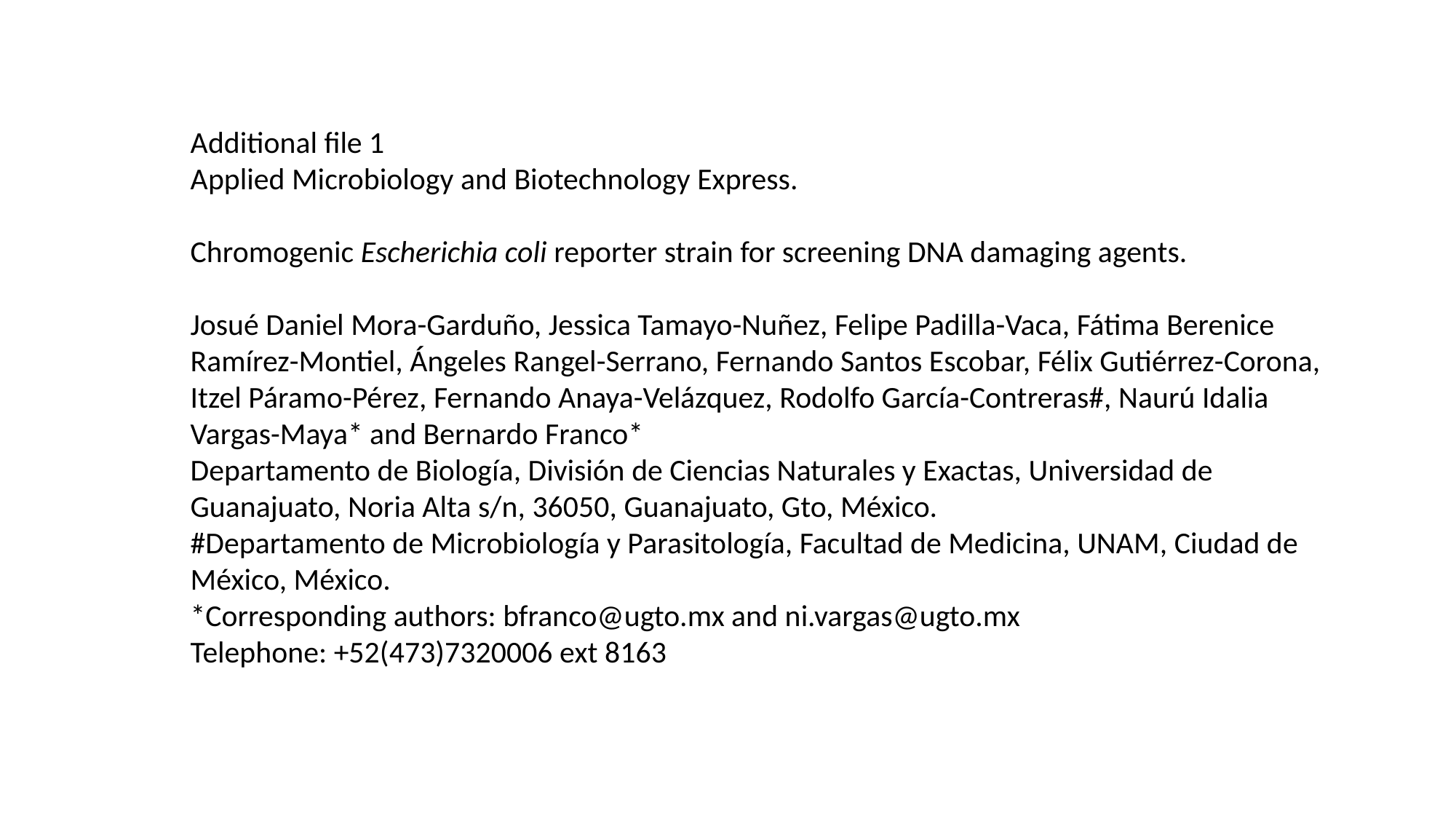

Additional file 1
Applied Microbiology and Biotechnology Express.
Chromogenic Escherichia coli reporter strain for screening DNA damaging agents.
Josué Daniel Mora-Garduño, Jessica Tamayo-Nuñez, Felipe Padilla-Vaca, Fátima Berenice Ramírez-Montiel, Ángeles Rangel-Serrano, Fernando Santos Escobar, Félix Gutiérrez-Corona, Itzel Páramo-Pérez, Fernando Anaya-Velázquez, Rodolfo García-Contreras#, Naurú Idalia Vargas-Maya* and Bernardo Franco*
Departamento de Biología, División de Ciencias Naturales y Exactas, Universidad de Guanajuato, Noria Alta s/n, 36050, Guanajuato, Gto, México.
#Departamento de Microbiología y Parasitología, Facultad de Medicina, UNAM, Ciudad de México, México.
*Corresponding authors: bfranco@ugto.mx and ni.vargas@ugto.mx
Telephone: +52(473)7320006 ext 8163

## Slide 2
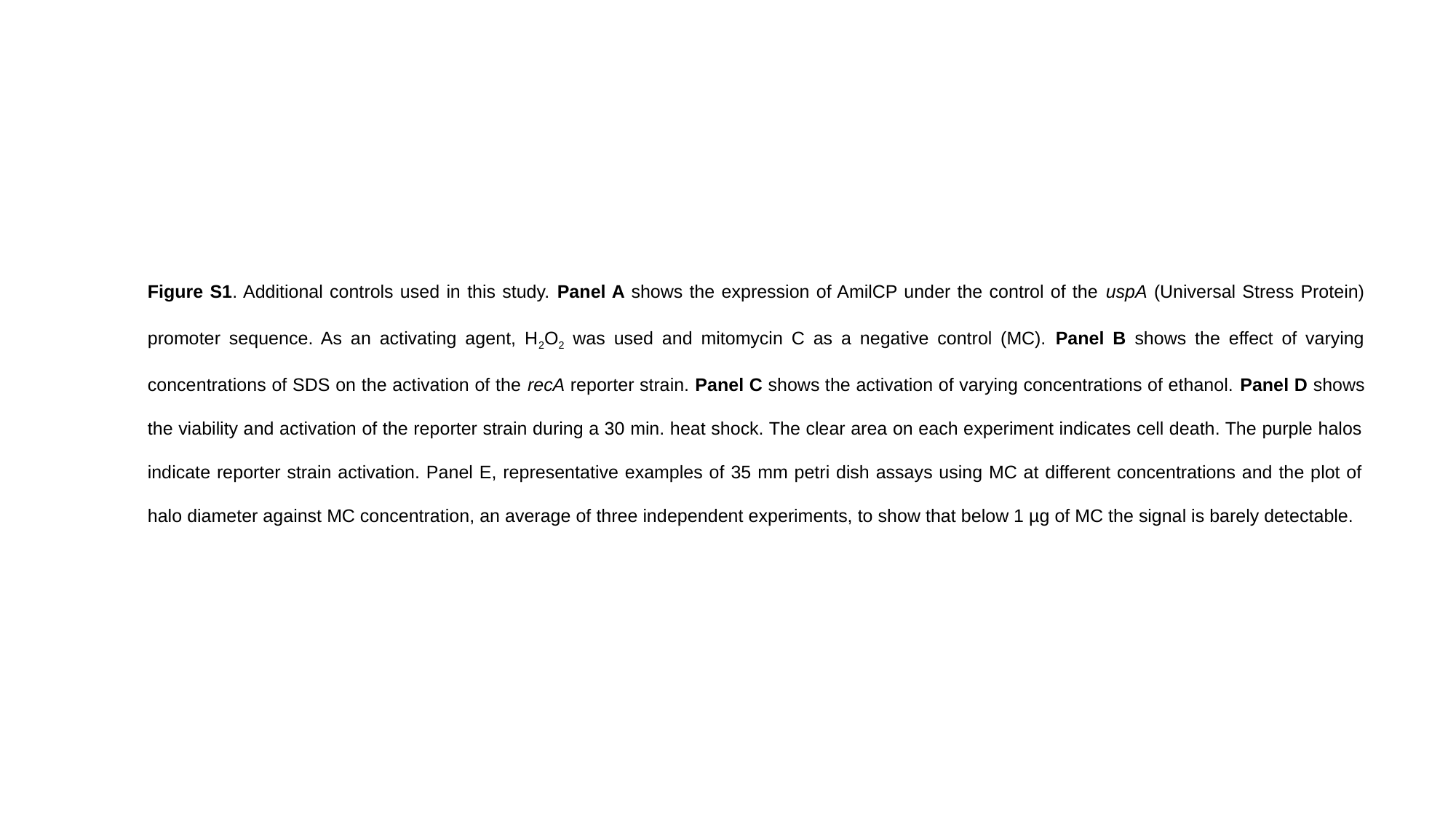

Figure S1. Additional controls used in this study. Panel A shows the expression of AmilCP under the control of the uspA (Universal Stress Protein) promoter sequence. As an activating agent, H2O2 was used and mitomycin C as a negative control (MC). Panel B shows the effect of varying concentrations of SDS on the activation of the recA reporter strain. Panel C shows the activation of varying concentrations of ethanol. Panel D shows the viability and activation of the reporter strain during a 30 min. heat shock. The clear area on each experiment indicates cell death. The purple halos indicate reporter strain activation. Panel E, representative examples of 35 mm petri dish assays using MC at different concentrations and the plot of halo diameter against MC concentration, an average of three independent experiments, to show that below 1 µg of MC the signal is barely detectable.

## Slide 3
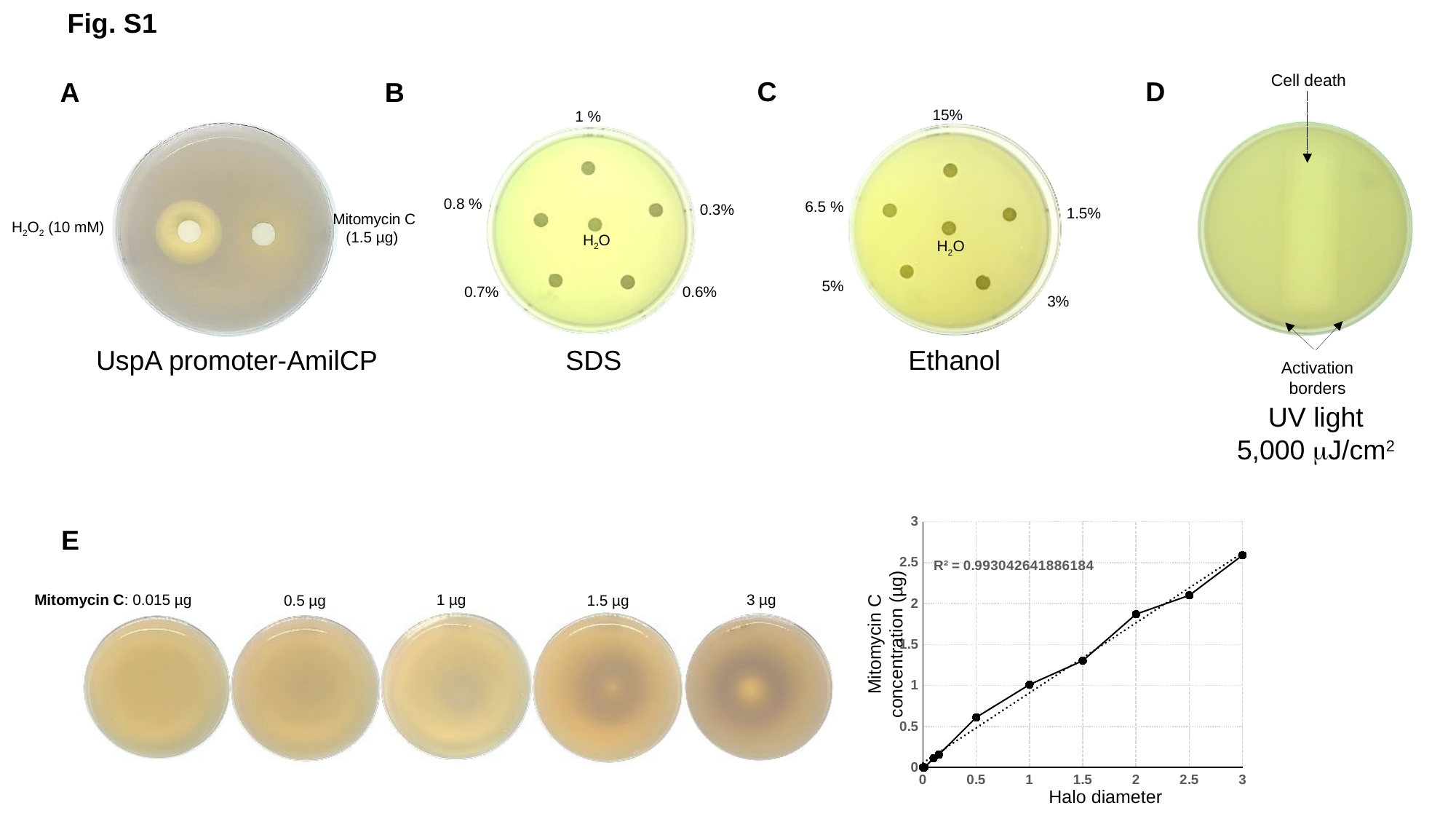

Fig. S1
Cell death
C
D
A
B
15%
6.5 %
1.5%
H2O
5%
3%
Ethanol
1 %
0.8 %
0.3%
H2O
0.7%
0.6%
SDS
 Mitomycin C
(1.5 µg)
H2O2 (10 mM)
UspA promoter-AmilCP
Activation borders
UV light
5,000 J/cm2
### Chart
| Category | Halo diameter |
|---|---|E
Mitomycin C: 0.015 µg
1 µg
3 µg
0.5 µg
1.5 µg
Mitomycin C concentration (µg)
Halo diameter

## Slide 4
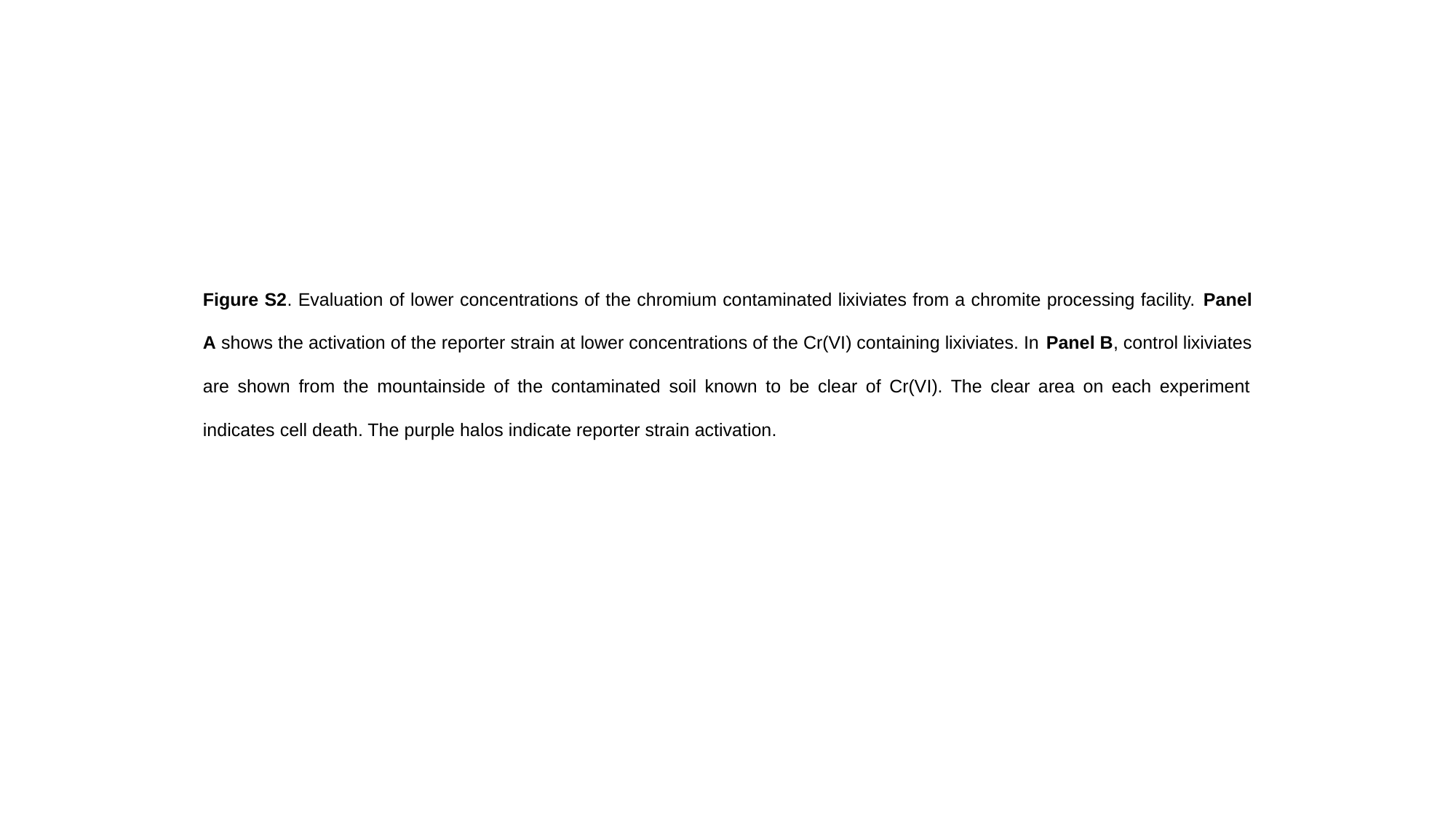

Figure S2. Evaluation of lower concentrations of the chromium contaminated lixiviates from a chromite processing facility. Panel A shows the activation of the reporter strain at lower concentrations of the Cr(VI) containing lixiviates. In Panel B, control lixiviates are shown from the mountainside of the contaminated soil known to be clear of Cr(VI). The clear area on each experiment indicates cell death. The purple halos indicate reporter strain activation.

## Slide 5
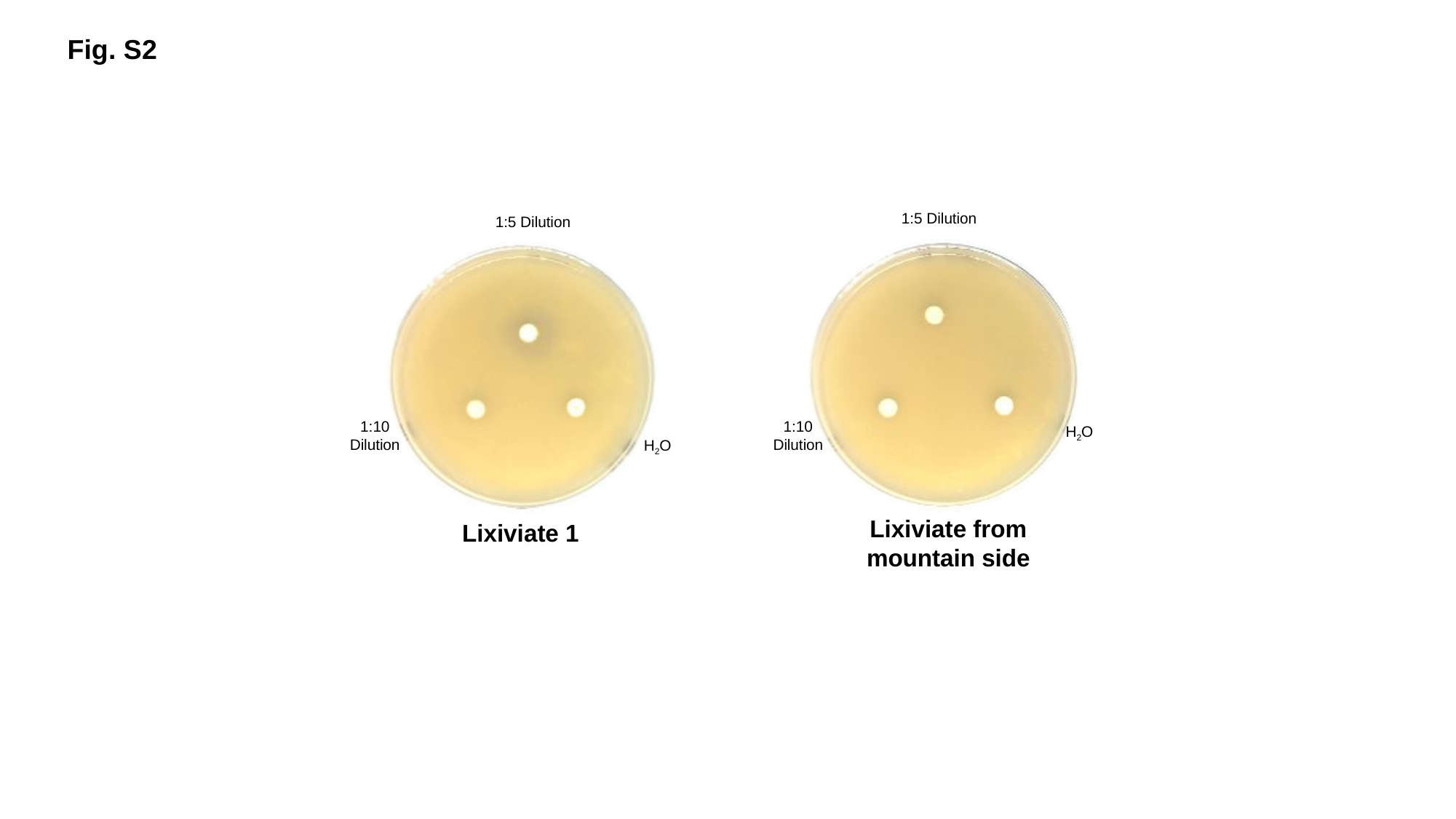

Fig. S2
1:5 Dilution
1:5 Dilution
H2O
Lixiviate 1
H2O
Lixiviate from mountain side
1:10 Dilution
1:10 Dilution
